# Factors Associated With Pregnancy and Childbirth Among Married Couples: A Longitudinal Analysis Using the 2012–2018 Korean Health Panel

**DOI:** 10.7759/cureus.71969

**Published:** 2024-10-20

**Authors:** Juhyeon Moon, Boyoung Jeon

**Affiliations:** 1 Graduate School of Public Health, Seoul National University, Seoul, KOR; 2 Department of Health and Medical Information, Myongji College, Seoul, KOR

**Keywords:** childbirth, lifestyle, longitudinal studies, maternal age, paternal age, pregnancy, reproductive health

## Abstract

Introduction: South Korea is experiencing a demographic paradox of the lowest birth rate worldwide with the longest life expectancy. Many studies on pregnancy and childbirth primarily focused on women's factors, often overlooking the contribution of both spouses. However, men also play a vital role in creating an environment for bearing and raising children. In addition, pregnancy and childbirth are considered part of family formation, based on decisions made by the couple. Therefore, this study aimed to assess factors influencing pregnancy and childbirth in married women of childbearing age and their spouses using seven years of representative survey data from South Korea.

Methods: A total of 2,579 married women aged 20-49 years and their spouses were identified in the 2012-2018 Korean Health Panel (KHP). The outcome variables were pregnancy and childbirth. The generalized estimating equation model was adopted using covariates of women's and men’s characteristics, comprising age, education, economic activity, smoking, drinking, physical activity, and body mass index (BMI), alongside having a family with children and income level as couple’s characteristics.

Results: In 2012-2017, the annual cases of pregnancies and childbirth were 1.43% and 1.02% in women aged ≥35 years vs. 17.76% and 10.81% in women aged <35 years, among married women identified in the KHP. Maternal and paternal age was the prominent factor: the adjusted odds ratio (aOR) of giving childbirth was 0.23 (95% confidence interval (CI): 0.15-0.29) for women aged ≥35 years and 0.39 (95% CI: 0.27-0.54) for men compared to those aged <35 years. Childbirth was more likely to occur in women with higher education and childless families. In the sub-analysis, women’s factors (e.g., alcohol consumption and infertility-related disease) and household income were more significant for women <35 years, but men’s factors (e.g., BMI and physical activity) were more significant for women aged ≥35 years.

Conclusions: Our findings presented only partial and heterogeneous relations regarding pregnancy and childbirth, unlike many biomedical and clinical studies emphasizing age, BMI, or health behaviors as fertility indicators. Fertility might be influenced not only by biological factors but also by socioeconomic stability and both women's and men’s factors, requiring caution in policy intervention.

## Introduction

South Korea is experiencing a demographic paradox because Korean women have the longest life expectancy and the lowest birth rate worldwide [1]. The rapid socioeconomic growth and changes in lifestyle in recent decades decreased the total fertility rate from 1.30 in 2012 to 0.72 in 2023 [2], with decreasing childbirth rate from 484,600 to 249,200 between 2012 and 2022. The reduction in childbirth was accompanied by the increased average age of first childbirth (31.6 years in 2012 vs. 33.5 years in 2022) and the increased percentage of mothers aged >35 years (18.7% in 2012 vs. 35.7% in 2022) [2]. Pregnancy in women of older age has become more prevalent over the last few decades. Women aged ≥35 years, commonly referred to as “advanced maternal age” [3], are at risk of maternal complications, including gestational diabetes mellitus, preeclampsia, postpartum depression, and bleeding, as well as negative fetal consequences, such as abruptio placentae, placenta previa, miscarriage, stillbirth, lower birth weight, premature birth, and chromosomal abnormalities [4, 5]. Therefore, considering the advanced maternal age in the analysis of factors associated with pregnancy and childbirth is necessary.

Moreover, investigating both indicators of pregnancy and childbirth is necessary because the two indicators represent the interconnected stages of reproductive health. For instance, pregnancy includes the period from conception to birth, while childbirth occurs at the end of pregnancy. Furthermore, not all pregnant women are guaranteed to give birth. Therefore, using data on both pregnancy and childbirth allows for more robust results to address reproductive health-related challenges [6].

Poor health and risky behavior of women affect pregnancy and childbirth [7, 8]. The World Health Organization established the following preconception priorities: healthy diet and nutrition, weight management, physical activity, planned pregnancy, and physical, mental, and psychosocial health [9-11]. Many of these priorities are connected to lifestyle, which can be challenging to change to improve fertility despite the awareness of the negative impacts [8]. The association between lifestyle and fertility is evidenced in men and women; hence, understanding the mechanisms underlying the impact of modifiable lifestyle behaviors is important [12]. For example, obesity or high waist circumference, poor diet, inactivity, smoking, excessive alcohol consumption, stress, and aging significantly contribute to the global decline in men's reproductive health [13, 14].

Although multiple lifestyle factors in both women and men contribute to reproductive health and pregnancy, studies conducted thus far primarily focused on the association between women's lifestyle and pregnancy. Additionally, research that considered the influence of couples' lifestyle factors is scarce [15]. Therefore, this study aimed to assess factors associated with pregnancy and childbirth in married women and men. Furthermore, this study showed differences based on maternal age by adopting the subgroup analysis and considering whether women are at an advanced maternal age or not.

## Materials and methods

Data source

The longitudinal household and individual data obtained between 2012 and 2018 were derived from the Korean Health Panel (KHP ver.1.7), which is jointly managed by the Korea Institute of Health and Social Affairs (KIHASA) and the National Health Insurance Service (NHIS) [16]. The KHP has the advantage of possessing concrete health information (including diagnosis, providers, and payments) according to medical service usage, with additional self-response health behavior information [17].

Variables

This study measured independent variables each year (observation T), with dependent variables created using data in the following year (observation T+1). Dependent variables determined whether women experienced pregnancy or childbirth one year after the observation year using a three-digit Korean Standard Classification of Diseases (KCD)-7 code. KCD-7 codes related to pregnancy and childbirth were defined with advice from a clinical expert working at a national hospital. Cases of pregnancy were defined as those who had visited outpatient clinics at least once for prenatal care (codes Z33, Z34, Z35, and Z36), while cases of childbirth were defined as those who had been admitted at least once for labor and delivery (codes O60, O64-O69, and O80-O84; Appendix 1). In this study, we do not distinguish between spontaneous pregnancy and assisted reproductive technology.

Independent variables at each observation were created using women's and men's characteristics, including age, education, economic activity, smoking, drinking, physical activity, and body mass index (BMI), as well as household characteristics, such as having a family with children and household income. Education was categorized as "high school or less" and "college or higher degree." Women's and men's economic activity differed, considering gendered labor market participation [18, 19]. Women's economic activity was categorized into three groups: unemployed, full-time, and part-time or self-employed, while men's economic activity was divided into two categories: full-time and others (unemployed, part-time, and self-employed). Health behavior variables, such as smoking, alcohol consumption, and physical activity, were included using self-reported questions. Smoking was measured as "currently smoking" or not, and alcohol consumption was categorized as "none, at least once a month, and at least once a week." Physical activity was categorized as "none, at least once or twice a week, and more than three times a week." The definition of physical activity was based on participation in vigorous physical activity or moderate physical activity. Vigorous physical activity was defined as "when you performed more than 10 minutes of physical activity that makes you breathe a lot and your heart rate increases a lot." Moderate physical activity was defined as "when it makes you breathe a little bit and your heart rate increases a little." The BMI threshold was 25, according to the Korean Society for the Study of Obesity guidelines [20, 21]. Family with children and household income, which influence family planning, were included as variables of family characteristics. The variable of a family having children was determined by whether the couple had one or more offspring. Five income quintiles were used to assess household income: 1st-3rd quintile represented low income, 4th quintile was middle income, and 5th quintile was high income, with the high-income group set as the reference group [20]. Additionally, female infertility-related diseases that had been diagnosed were coded as binary independent variables to control the pathologic condition.

Study participants

**Figure 1:**
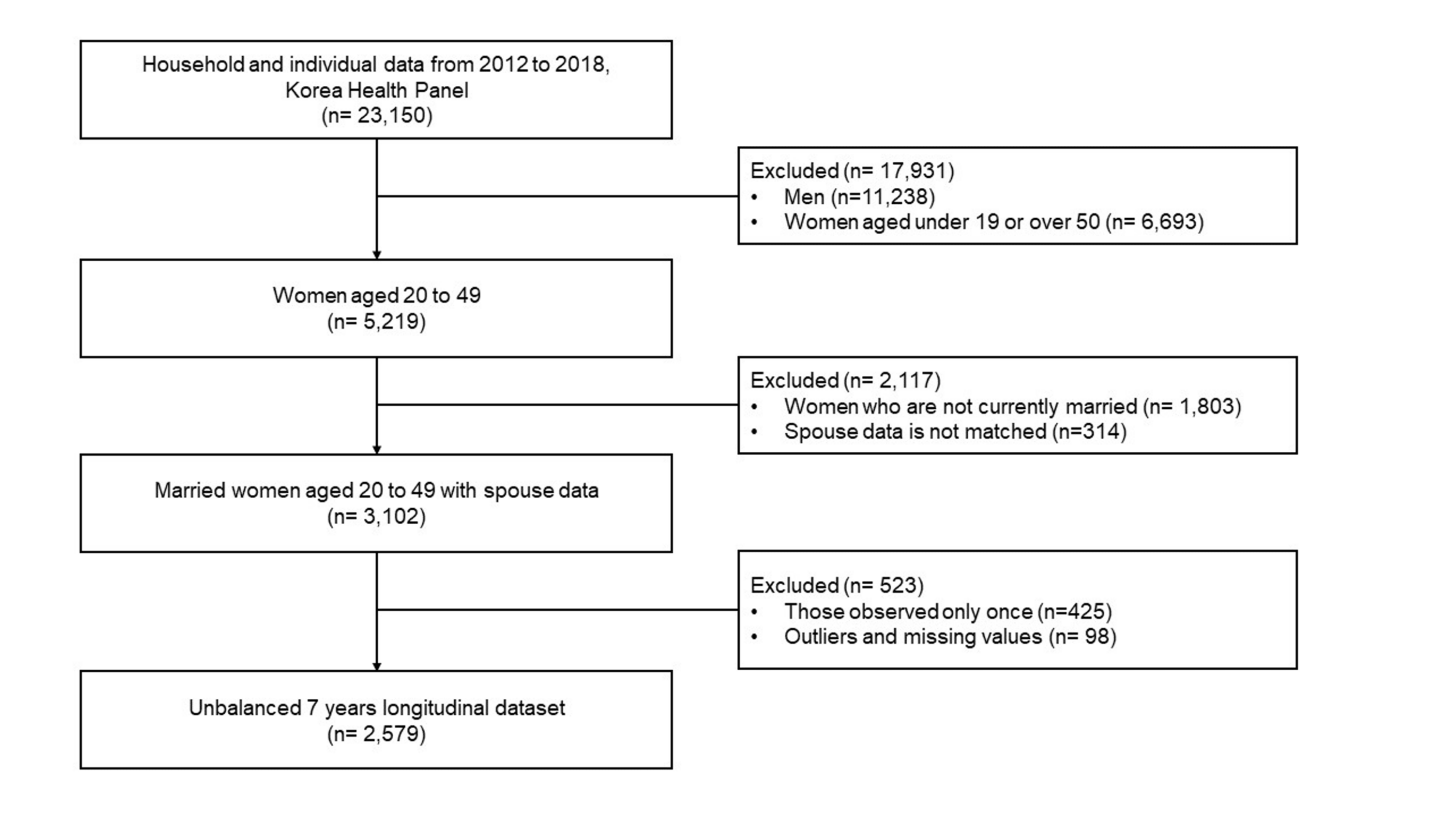
Study participants

Married women aged 20-49 years were identified among the 23,150 individuals in the raw data (Figure 1). Characteristics such as sex, age, marital status, household structure (whether the household members are composed of the first, second, third, or other generations), and family relationship (the relationship between the household head and the household members who participated in the survey) were used to merge the spouses' information. A total of 5,219 women aged 20-49 years participated in the survey during the observation period, among whom, 3,102 women who were married at each observation and whose spouse's information was matched were selected. No men were diagnosed with male infertility (N46: male infertility, azoospermia NOS) in outpatient and inpatient care (n = 0). Those who were observed for only one year (n = 425) and participants with any outliers or missing values (n = 98) were excluded. The final number of study participants was 2,579 (11,216 observations for seven years) based on women household members.

Statistical analysis

The annual trends in the number of participants and the number and percentage of women who experienced pregnancy and childbirth in the following year were compared before and after the age of 35 years. During the first observation, the general characteristics of covariates according to childbirth were tested using chi-square analysis.

This study applied the generalized estimating equation (GEE) model to address problems of correlation due to a subject's repeated measurements in the longitudinal datasets. The GEE model was conducted to estimate the population-average effects of factors influencing pregnancy and childbirth for married women, accounting for the correlation within the subject over time, providing robust estimates of the association between predictors and outcomes even with repeated measures [22]. The adjusted odds ratio (aOR) and 95% confidence intervals (CI) were calculated for time-varying covariates of women, men, and households. Finally, a sub-analysis was performed to compare differences in women's age before and after 35 years, considering their average age at their first marriage and first experience of childbirth. All statistical analyses were performed using SAS 9.4 (SAS Inc., Cary, NC). This secondary analysis underwent an ethics review by the Korea National Institute for Bioethics Policy (IRB number: P01-202208-01-025).

## Results

Pregnancy and childbirth trends

Table 1 shows the annual observations and cases of pregnancy and childbirth in the following year in women aged <35 and ≥35 years. In 2012-2017, the annual cases of pregnancies and childbirth were 1.43% and 1.02% in women aged ≥35 years vs. 17.76% and 10.81% in women aged <35 years, respectively. 

**Table 1:** The number and rate of married women of childbearing age who have experienced pregnancy and childbirth before and after the age of 35 years (2012–2017)

Variables	2012	2013	2014	2015	2016	2017	Total
Women aged <35 years	323	303	363	328	273	223	1,813
Pregnancy in the next year	52 (16.10)	63 (20.79)	66 (18.18)	58 (17.68)	41 (15.02)	42 (18.83)	322 (17.76)
Childbirth in the next year	31 (9.60)	36 (11.88)	40 (11.02)	34 (10.37)	35 (12.82)	20 (8.97)	196 (10.81)
Women aged ≥35 years	1,378	1,459	1,792	1,698	1,573	1,503	9,403
Pregnancy in the next year	14 (1.02)	17 (1.17)	31 (1.73)	23 (1.35)	22 (1.40)	27 (1.80)	134 (1.43)
Childbirth in the next year	16 (1.16)	11 (0.75)	23 (1.28)	12 (0.71)	16 (1.02)	18 (1.20)	96 (1.02)
Total	1,701	1,762	2,155	2,026	1,846	1,726	11,216

General characteristics of the study population

In the baseline year of 2012, the number of study participants was 2,579. General characteristics showed whether there was a difference according to childbirth experience in the following year (Table 2). Factors that were statistically significant at a 5% alpha level were women’s age, education, and economic activity; men’s age and economic activity; and presence or absence of children in a family. Additionally, health- and healthy lifestyle-related factors, such as smoking, drinking, moderate-to-severe physical activity, BMI, and infertility-related diseases, did not differ. Among men whose wives experienced childbirth, 47.79% reported drinking alcohol at least once a week, compared to 59.25% of men whose wives did not give birth (p < 0.05). However, half of men were current smokers regardless of the presence of children.

**Table 2:** Comparison of the first-year observations depending on whether women experienced childbirth in the following year (N = 2,579) *p < 0.05, **p < 0.01, ***p < 0.001

Variables		No	Yes	χ2 and p-value
Women’s characteristics		N (%)	N (%)	
Age (years)	<35	556 (22.55)	82 (72.57)	χ2 = 145.1976***
	≥35	1,910 (77.45)	31 (27.43)	
Education	High school or less	1,285 (52.11)	30 (26.55)	χ2 = 28.2470***
	College or higher	1,181 (47.89)	83 (73.45)	
Economic activity	Unemployed	1,092 (44.28)	53 (46.90)	χ2 = 18.8498***
	Full-time employed	486 (19.71)	38 (33.63)	
	Part-time or self-employed	1888 (36.01)	22 (19.47)	
Smoking	No smoker	2425 (98.34)	111 (98.23)	χ2 = 0.0076
	Current smoker	41 (1.66)	2 (1.7)	
Alcohol consumption	None	803 (32.56)	33 (28.32)	χ2 = 1.5807
	At least once a month	1,119 (45.38)	51 (45.13)	
	At least once a week	544 (22.06)	30 (26.55)	
Moderate-to-high physical activity	None	1,569 (63.63)	77 (68.14)	χ2 = 4.0396
	At least once or twice a week	246 (9.98)	15 (13.27)	
	More than three times a week	651 (26.40)	21 (18.58)	
BMI	<25	2,041 (82.77)	92 (81.42)	χ2 = 0.1376
	≥25	425 (17.23)	21 (18.58)	
Infertility-related disease	None	2,089 (84.71)	96 (84.96)	χ2 = 0.0050
	Diagnosed	377 (15.29)	17 (15.04)	
Couples' characteristics				
Family with children	Have children	2,240 (90.84)	61 (53.98)	χ2 = 152.5838***
	No children	226 (9.16)	52 (46.02)	
Income level	Low income	498 (20.19)	18 (15.93)	χ2 = 2.9964
	Middle income	1288 (52.23)	56 (49.56)	
	High income	680 (27.58)	39 (34.51)	
Husbands' characteristics				
Age (years)	<35	332 (13.46)	73 (64.60)	χ2 = 213.4554***
	≥35	2,134 (86.54)	40 (35.40)	
Education	High school or less	1,070 (43.39)	38 (33.63)	χ2 = 4.2017*
	College or higher	1,396 (56.61)	75 (66.37)	
Economic activity	Full-time employed	1,328 (53.85)	83 (73.45)	χ2 = 16.7501***
	Others (unemployed, part-time, and self-employed)	1,138 (46.15)	30 (26.55)	
Smoking	Non-smoker	1,224 (49.64)	57 (50.44)	χ2 = 0.0282
	Current smoker	1242 (50.36)	56 (49.56)	
Alcohol consumption	None	256 (10.38)	10 (8.85)	χ2 = 8.5527*
	At least once a month	749 (30.37)	49 (43.36)	
	At least once a week	1461 (59.25)	54 (47.79)	
Moderate-to-high physical activity	None	1,010 (40.96)	51 (45.13)	χ2 = 1.3907
	At least once or twice a week	495 (20.07)	18 (15.93)	
	More than three times a week	961 (38.97)	44 (38.94)	
BMI	<25	1,590 (64.48)	68 (60.18)	χ2 = 0.8702
	≥25	876 (35.52)	45 (39.82)	
N		2,466 (100.0)	113 (100.0)	

Factors associated with pregnancy and childbirth in the following year

Table 3 demonstrates that both men's and women's ages, women’s levels of education and economic activity, as well as drinking behavior, had a significant average effect on both pregnancy and childbirth in the following year among women aged 20-49 years. For women, the age ≥35 years were characterized by a decreased likelihood of pregnancy (aOR: 0.21, 95% CI: 0.15-0.29) and childbirth (aOR: 0.23, 95% CI: 0.16-0.32) compared to that at age <35 years. Similarly, men’s age of ≥35 years was characterized by a decreased likelihood of their partner's experiencing pregnancy (aOR: 0.34, 95% CI: 0.25-0.45) and childbirth (0.39, 95% CI: 0.27-0.54) compared to that at age <35 years. Among families who already had children, the likelihood of pregnancy (aOR: 0.41, 95% CI: 0.30-0.54) and childbirth (aOR: 0.38, 95% CI: 0.27-0.51) were lower compared to families without children. Higher education, with a college or university degree, was associated with a higher likelihood of pregnancy (aOR: 1.89, 95% CI: 1.36-2.60) and childbirth (aOR: 1.89, 95% CI: 1.35-2.62) compared to women with education of high school or less. Regarding economic activity, women who had full-time jobs had a lower likelihood of pregnancy (aOR: 0.76, 95% CI: 0.56-1.02) and those with part-time jobs or self-employed had a lower likelihood of childbirth (aOR: 0.71, 95% CI: 0.51-0.99) compared to unemployed women. Moreover, men employed full-time were more likely to have partners who gave birth (aOR: 1.45, 95% CI: 1.08-1.94), compared to those who were unemployed, working part-time, or self-employed. Among women’s health-related factors, alcohol consumption at least once a week affected both pregnancy and childbirth, and women’s infertility-related diseases had a positive relation with pregnancy. Furthermore, a man’s BMI of ≥25 was positively associated with the likelihood of their partners experiencing childbirth.

**Table 3:** Longitudinal analysis of factors influencing pregnancy and childbirth in the following year among married couples in the Korean Health Panel (2012−2017) Abbreviations: ref., reference; aOR, adjusted odd ratio; CI, confidence interval *p < 0.1, **p < 0.05, ***p < 0.01

Variables		Pregnancy		Childbirth	
		aOR	(95% CI)	aOR	(95% CI)
Women’s characteristics					
Age, years (ref. <35)	≥35	0.21***	(0.15, 0.29)	0.23***	(0.16, 0.32)
Education (ref. high school or less)	College or higher	1.89***	(1.36, 2.60)	1.89***	(1.35, 2.62)
Economic activity (ref. unemployed)	Full-time employed	0.76*	(0.56, 1.02)	0.76	(0.55, 1.05)
	Part-time or self-employed	0.80	(0.60, 1.06)	0.71**	(0.51, 0.99)
Smoking (ref. non-smoker)	Current smoker	1.44	(0.64, 3.19)	1.33	(0.53, 3.27)
Alcohol consumption (ref. none)	At least once a month	1.03	(0.79, 1.32)	1.11	(0.82, 1.49)
	At least once a week	1.35*	(0.99, 1.83)	1.49**	(1.04, 2.11)
Physical activity (ref. none)	At least once or twice a week	1.26	(0.90, 1.76)	1.13	(0.75, 1.71)
	More than three times a week	0.93	(0.71, 1.21)	0.85	(0.61, 1.18)
BMI (ref. <25)	≥25	0.99	(0.72, 1.35)	1.08	(0.77, 1.50)
Infertility-related disease (ref. not applicable)	Diagnosed	1.32**	(1.00, 1.73)	1.03	(0.74, 1.44)
Couple’s characteristics					
Family structure (ref. no children)	Have children	0.41***	(0.30, 0.54)	0.38***	(0.27, 0.51)
Household income	Low income	0.79	(0.54, 1.14)	0.90	(0.58, 1.37)
(ref. high income)	Middle income	0.93	(0.71, 1.21)	1.09	(0.80, 1.47)
Husbands' characteristics					
Age, years (ref. <35)	≥35	0.34***	(0.25, 0.45)	0.39***	(0.27, 0.54)
Education (ref. high school or less)	College or higher	1.05	(0.77, 1.41)	0.96	(0.70, 1.30)
Economic activity (ref. others: unemployed, part-time, and self-employed)	Full-time employed	1.21	(0.93, 1.56)	1.45**	(1.08, 1.94)
Smoking (ref. non-smoker)	Current smoker	0.92	(0.72, 1.17)	1.00	(0.77, 1.29)
Alcohol consumption (ref. none)	At least once a month	1.40	(0.90, 2.16)	1.06	(0.67, 1.66)
	At least once a week	1.22	(0.78, 1.89)	0.85	(0.54, 1.32)
Physical activity (ref. none)	At least once or twice a week	0.98	(0.74, 1.30)	0.85	(0.59, 1.19)
	More than three times a week	0.91	(0.71, 1.15)	0.86	(0.64, 1.14)
BMI (ref. <25)	≥25	1.04	(0.81, 1.30)	1.30**	(1.01, 1.66)
Year (ref. 2012)	2013	1.23	(0.88, 1.70)	0.99	(0.64, 1.53)
	2014	1.16	(0.84, 1.60)	1.09	(0.72, 1.63)
	2015	0.95	(0.67, 1.33)	0.81	(0.52, 1.25)
	2016	0.84	(0.58, 1.20)	1.08	(0.70, 1.65)
	2017	1.04	(0.72, 1.49)	0.84	(0.52, 1.32)
N		11,216		11,216	

Comparing the groups of women aged <35 and ≥35 years

Subgroup analysis was conducted according to the women’s age of 35 years, excluding the educational variable, which does not tend to change after the age of 30 years (Table 4). The average effect of families with children and men’s age was firmly maintained in both subgroups. Women’s alcohol consumption was positively associated with childbirth (aOR: 1.57, 95% CI: 1.03-2.39) only in the younger group. Infertility-related disease remained positively significant in the younger group for pregnancy (aOR: 1.40, 95% CI: 1.00-1.95). Furthermore, low household income significantly reduced the likelihood of pregnancy (aOR: 0.57, 95% CI: 0.36-0.87) and childbirth (aOR: 0.55 95% CI: 0.33-0.90) compared to high-income households among women aged <35 years. In the group of women aged ≥35 years, alcohol consumption and income level lost statistical significance. Instead, the effects of men’s economic status increased the odds of pregnancy and childbirth. Additionally, higher BMI in men was associated with an increased likelihood of their partners experiencing childbirth, while participation in physical activity among men was linked to a decreased chance of pregnancy and childbirth for women aged ≥35 years.

**Table 4:** Subgroup analysis by women's age: Longitudinal analysis of factors influencing pregnancy and childbirth in the following year among married couples in the Korean Health Panel (2012−2017) In this table, the education variable was excluded from the subgroup analysis. Abbreviations: ref., reference; aOR, adjusted odd ratio; CI, confidence interval. *p < 0.1, **p < 0.05, ***p < 0.01

Characteristics		Pregnancy		Childbirth	
		<35 years	≥35 years	<35 years	≥35 years
Women's characteristics		aOR (95% CI)	aOR (95% CI)	aOR (95% CI)	aOR (95% CI)
Economic activity (ref. unemployed)	Full-time employed	0.84	0.71	0.79	0.78
		(0.59, 1.19)	(0.42, 1.19)	(0.53, 1.15)	(0.44, 1.36)
	Part-time or self-employed	0.9	0.73	0.78	0.63
		(0.61, 1.29)	(0.47, 1.11)	(0.51, 1.20)	(0.38, 1.04)
Smoking (ref. non-smoker)	Current smoker	1.34	1.42	1.25	0.53
		(0.56, 3.19)	(0.41, 4.94)	(0.47, 3.28)	(0.06, 4.14)
Alcohol consumption (ref. none)	At least once a month	1.08	0.97	1.18	0.91
		(0.80, 1.48)	(0.62, 1.51)	(0.82, 1.67)	(0.54, 1.50)
	At least once a week	1.42*	1.09	1.57**	1.18
		(0.98, 2.04)	(0.63, 1.87)	(1.03, 2.39)	(0.64, 2.13)
Physical activity (ref. none)	At least once or twice a week	1.24	1.38	1.05	1.39
		(0.79, 1.92)	(0.79, 2.38)	(0.61, 1.78)	(0.71, 2.68)
	More than three times a week	0.83	1.26	0.76	1.14
		(0.58, 1.17)	(0.83, 1.90)	(0.49, 1.15)	(0.68, 1.88)
BMI (ref. <25)	≥25	1.01	0.74	1.14	0.7
		(0.70, 1.45)	(0.43, 1.25)	(0.78, 1.66)	(0.38, 1.25)
Infertility-related disease (ref. not applicable)	Diagnosed	1.40**	1.27	0.98	1.08
		(1.00, 1.95)	(0.81, 1.96)	(0.65, 1.48)	(0.62, 1.84)
Couple’s characteristics					
Family with children (ref. no children)	Have children	0.51***	0.15***	0.56***	0.13***
		(0.36, 0.70)	(0.09, 0.25)	(0.39, 0.80)	(0.07, 0.22)
Income level (ref. high income)	Low income	0.57**	1.22	0.55**	1.72
		(0.36, 0.87)	(0.64, 2.27)	(0.33, 0.90)	(0.82, 3.60)
	Middle income	0.76	1.06	0.79	1.48
		(0.54, 1.06)	(0.69, 1.63)	(0.54, 1.14)	(0.88, 2.47)
Husbands' characteristics					
Age, years (ref. <35)	≥35	0.37***	0.14***	0.46***	0.12***
		(0.27, 0.49)	(0.06, 0.27)	(0.34, 0.63)	(0.05, 0.23)
Economic activity (ref. others: unemployed, part-time, and self-employed)	Full-time employed	1.19	1.62**	1.35*	2.08***
		(0.87, 1.62)	(1.06, 2.46)	(0.95, 1.89)	(1.28, 3.36)
Smoking (ref. non-smoker)	Current smoker	0.83	0.98	0.82	1.19
		(0.62, 1.10)	(0.66, 1.45)	(0.60, 1.09)	(0.76, 1.82)
Drinking (ref. none)	At least once a month	1.08	1.58	0.89	1.44
		(0.64, 1.78)	(0.79, 3.11)	(0.52, 1.50)	(0.65, 3.15)
	At least once a week	1.07	0.98	0.76	0.99
		(0.64, 1.77)	(0.48, 1.95)	(0.45, 1.28)	(0.45, 2.16)
Physical activity (ref. none)	At least once or twice a week	1.21	0.71	1.07	0.57*
		(0.84, 1.72)	(0.44, 1.15)	(0.70, 1.62)	(0.30, 1.07)
	More than three times a week	1.17	0.61**	1	0.79
		(0.87, 1.57)	(0.40, 0.92)	(0.70, 1.41)	(0.48, 1.26)
BMI (ref. <25)	≥25	1.06	1.26	1.1	1.74**
		(0.80, 1.40)	(0.85, 1.85)	(0.81, 1.47)	(1.13, 2.65)
Year (ref. 2012)	2013	1.28	1.17	1.27	0.69
		(0.85, 1.90)	(0.63, 2.14)	(0.74, 2.16)	(0.31, 1.47)
	2014	1.08	1.42	1.16	1.05
		(0.72, 1.61)	(0.81, 2.48)	(0.69, 1.95)	(0.54, 1.99)
	2015	0.95	0.97	1.02	0.54
		(0.62, 1.43)	(0.52, 1.77)	(0.59, 1.74)	(0.25, 1.15)
	2016	0.8	0.95	1.38	0.77
		(0.51, 1.26)	(0.50, 1.76)	(0.81, 2.34)	(0.37, 1.57)
	2017	1	1.16	0.9	0.86
		(0.63, 1.59)	(0.63, 2.12)	(0.49, 1.64)	(0.42, 1.71)
N		1,813	9,403	1,813	9,403

## Discussion

In this study, we examined the average effect of factors associated with pregnancy and childbirth in married women and their spouses. In 2012-2017, the annual cases of pregnancies and childbirth were 1.43% and 1.02% in women aged ≥35 years vs. 17.76% and 10.81% in women aged <35 years, among 2,579 married women identified in the KHP. The result of this study could be considered representative of the target population. As of 2015, the percentage of children born by married women aged 20-34 years was 10.15%, and the percentage of children born by married women aged 35 to 49 was 1.64% in the Korean national statistics [2]. We performed further analysis by dividing subgroups into age categories of <35 and ≥35 years given the rising average childbearing age among women. Women aged ≥35 years, men aged ≥35 years, and families already having children had a lower likelihood of pregnancy in the next year. Conversely, women’s higher education status, alcohol consumption, and infertility-related diseases had positive influences on the likelihood of pregnancy. Women who consumed alcohol at least once a week and those who were not economically active were more likely to experience childbirth. On the other hand, women aged ≥35 years, those in full-time employment, families with children, men aged aged ≥35 years, and men with insecure jobs all showed a negative relationship with childbirth. Moreover, women aged <35 years were prone to have a positive relation with drinking behavior and a negative effect of lower household income on family planning. For women aged ≥35 years, their husband's characteristics, including economic activity, physical activity, and BMI, were more strongly associated with the chance of pregnancy and childbirth than their own characteristics. Additionally, families with children and men’s age maintained statistical significance regardless of outcome measure and subgroup analysis.

Pregnancy at advanced maternal age (≥35 years) is considered a risk factor for adverse maternal and perinatal outcomes [5]. Many well-designed large studies and meta-analyses showed that advanced maternal age impacts maternal and perinatal adverse outcomes, decreasing fertility. Even when pregnancy is achieved, women of advanced maternal age and their physicians experience and manage maternal complications [3]. Additionally, women’s infertility-related diseases, such as deformities, dysfunction, diseases of reproductive organs, and hormonal imbalance, increased the possibility of pregnancy in the following year. This may be explained by the fact that women with a strong intention to have a child are more likely to visit their gynecologist and undergo health screenings, resulting in earlier detection of infertility-related diseases [20]. In Korea, visiting an obstetrician or gynecologist before pregnancy is uncommon; thus, the likelihood of being diagnosed with an infertility-related disease is lower if someone has no experience of pregnancy [23]. Therefore, the interpretation of the relationship between infertility-related diseases and pregnancy requires caution.

Women’s job status is a key factor in an individual’s pregnancy and childrearing [9]. When women have a full-time job or are self-employed, they tend to prioritize their professional careers at work [24]. Due to drastic improvements in women’s education and labor participation over the past few decades, highly educated women face challenges in the work-life balance [25]. Consequently, women are more likely to refuse or delay having children, reducing the proportion of households that have more than one child [18, 19, 26].

In our study, household income had a significant effect on pregnancy and childbirth in women aged <35 years, meaning that a higher financial capacity provides a higher willingness to have children [27]. The decline in the fertility rate among women of lower social classes has significantly contributed to the overall decrease in the total fertility rate in Korea [28]. Additionally, men’s economic activity had a more robust effect than women’s economic activity or household income. This robustness indicates that men are expected to play the role of the breadwinner, which necessitates full-time work [20]. On the other hand, low fertility rates are a social issue in European countries, where women tend to have more children when part-time work is well established, or when men have the option to reduce their working hours in the labor market [29].

In contrast to previous studies, women’s alcohol consumption (once a week) was positively associated with higher rates of pregnancy and childbirth among women aged <35 years. This is in contrast to findings that alcohol consumption can influence the risk of recurrent pregnancy loss [12]. Women’s alcohol consumption may be related to social participation. Caution is needed when interpreting the results of this study because this study cannot detect problematic drinking habits and account for overlapping periods with pregnancy.

Pregnancy and childbirth were influenced by both women's and men’s factors, including age, socioeconomic status, and lifestyles. Men's factors, such as aging, stress, nutrition, physical activity, and environmental factors, increase the risk of male infertility, similar to women’s factors affecting fertility [30]. Our study presents results opposite to those of previous studies [13, 14, 30, 31]. Men's higher BMI was positively associated with their wives' likelihood of childbirth, while men's regular physical activity had a negative association with pregnancy among women aged >35 years. The varying criteria for measuring physical activity intensity across studies may also lead to different outcomes. Additionally, the business dinners that men frequently attend, which often involve alcohol consumption, could contribute to the positive association between high BMI in men and childbirth [32, 33]. Thus, follow-up research on men’s factors is needed to determine the exact mechanism.

Limitations

Our study has some limitations. First, outcome variables did not contain fetal health information to determine the health outcome. Second, information regarding contraception was lacking due to data limitations and the fact that social health insurance does not cover contraceptive measures. Third, parents' fertility preference (e.g., number of children or preference for sons and daughters) may affect pregnancy and childbirth, although it could not be included in this study. Therefore, these limitations need to be considered in future studies. Moreover, this study could not adjust for the effects of men’s infertility because there were no men who used healthcare services or were diagnosed with male infertility. Hence, further studies need to consider men's infertility and related diseases. Finally, since this study selected a subsample of the KHP and applied inclusion and exclusion criteria during the sampling process, we did not use the statistical weights of the KHP. If we had applied the weights of the KHP, it would have increased the risk of bias because the study participants represented neither the entire Korean population nor the initial survey subjects. We confirmed the external validity of the results, rather than the weights of the KHP, by comparing the Korean national statistics.

## Conclusions

We estimated population-average effects related to pregnancy and childbirth among married women and men in 2012-2018. We found that women’s pregnancy and childbirth were associated with a complex combination of factors such as physical health, socioeconomic stability, and the influence of their partners. Among women aged <35 years, the impact of alcohol consumption, infertility-related diseases, and household income were significant factors for the likelihood of pregnancy or childbirth. In contrast, among women aged ≥35 years, men’s characteristics impacted the chance of childbirth. Therefore, pregnancy and childbirth are influenced not only by biological changes but also by socioeconomic stability and both women's and men’s factors, calling for caution in policy interventions.

## Appendices

 Appendix 1

Table 5 shows the Korean Standard Classification of Disease (KCD)-7 code and definitions for dependent variables.

**Table 5:** Korean Standard Classification of Diseases (KCD)-7 code and definitions for dependent variables

Dependent variables	KCD-7 code and definition
Pregnancy	Cases of pregnancy were defined as those who had visited outpatient clinics at least once for prenatal care (Z33, Z34, Z35, Z36). Z33: Pregnant state, incidental (Pregnant state NO); Z34: Supervision of normal pregnancy (Less than 24 weeks pregnant, 22 weeks pregnant or more, less than 34 weeks pregnant, 34 weeks or more pregnant, unspecified pregnancy duration); Z35: Supervision of high-risk pregnancy; Z36: Antenatal screening (Abnormal findings on antenatal screening of mother (O28.-, exclusion), Routine prenatal care(Z34-Z35, exclusion))
Childbirth	Cases of childbirth were defined as those who had been admitted at least once for labor and delivery (O60, O64-O69, O80-O84). O60: Preterm labor and delivery; O64: Obstructed labor due to malposition and malpresentation of fetus; O65: Obstructed labor due to maternal pelvic abnormality; O66: Other obstructed labor; O67: Labor and delivery complicated by intrapartum hemorrhage; NEC O68: Labor and delivery complicated by fetal stress [distress]; O69: Labor and delivery complicated by umbilical cord complications; O80: Single spontaneous delivery; O81: Single delivery by forceps and vacuum extractor; O82: Single delivery by caesarean section; O83: Other assisted single delivery; O84: Multiple deliveries
